# Supplementary material for: Universal inter-molecular radical transfer reactions on metal surfaces
Source: Nat Commun. 2024 Apr 8;15:3030. doi: 10.1038/s41467-024-47252-1 (PMC11001993; doi:10.1038/s41467-024-47252-1)
Supplement: Supplementary file 1 — Supplementary Information [file 41467_2024_47252_MOESM1_ESM.pdf]

Supplementary Information for

Universal inter-molecular radical transfer reactions on metal surfaces

Junbo Wang,[a,b,#] Kaifeng Niu,[b,c,#] Huaming Zhu,[a,#] Chaojie Xu,[b] Chuan Deng,[a] Wenchao Zhao,[d] Peipei Huang,[a] Haiping Lin,[a] Dengyuan Li,[e] Johanna Rosen,[c] Peinian Liu,[e] Francesco Allegretti,[d] Johannes V. Barth,[d] Biao Yang,[b,d,]* Jonas Björk,[c,]* Qing Li,[a,]* and Lifeng Chi,[b,f,]*

[a]School of Physics and Information Technology, Shaanxi Normal University, Xi'an 710119, China

[b]Institute of Functional Nano & Soft Materials (FUNSOM), Jiangsu Key Laboratory for Carbon-Based Functional Materials & Devices, Soochow University, Suzhou 215123, China

[c]Department of Physics, Chemistry and Biology, IFM, Linköping University, Linköping 58183, Sweden

[d]Physics Department E20, Technical University of Munich, James-Franck-Str. 1, 85748, Garching, Germany

[e]Key Laboratory for Advanced Materials and Feringa Nobel Prize Scientist Joint Research Center, Frontiers Science Center for Materiobiology and Dynamic Chemistry, State Key Laboratory of Chemical Engineering, School of Chemistry and Molecular Engineering, East China University of Science & Technology, Shanghai 200237, China

[f]Department of Materials Science and Engineering, Macau University of Science and Technology, Macau 999078, China

[#]These authors contributed equally.

*Email: biao.yang@tum.de, jonas.bjork@liu.se, liqing@snnu.edu.cn, chilf@suda.edu.cn

Suppl. Note 1. Reaction behavior of Ext-TEB and DBTP separately on Ag(111)

Supplementary Fig. 1a depicts a representative STM image after depositing 0.8 monolayer (ML) 1,3,5-tris-(4-ethynylphenyl) benzene (Ext-TEB, the structure model is shown in the inset of Supplementary Fig. 1a) on Ag(111) held at room temperature (RT). The precursor molecules self-assemble into large supramolecular domains (the structural model is shown in Supplementary Fig. 1b). The coupling of Ext-TEB takes place after annealing the sample at 400 K (Supplementary Fig. 1c).[1-4] The corresponding structural model is given in the Supplementary Fig. 1d.

The deposition of 4,4”-Dibromo-p-terphenyl (DBTP, structural model is shown in the inset of Supplementary Fig. 1e) on Ag(111) held at RT leads to the formation of two-dimensional porous network, through Br···Br bonds and Br···H bonds (Supplementary Fig. 1f). Regular propeller-like patterns are observed inside the hexagonal pores. This phenomenon is attributed to a caged molecule hopping around four stable adsorption configurations, facilitated by weak Br···Br and Br···H bond between the caged monomer and adjacent molecules. The detailed structural model can be seen in previous reports.[5,6] After annealing the sample to 370 K, the network was transformed into ladder organometallic oligomers (Supplementary Fig. 1g). Closer investigations reveal that the oligomers are formed by the Ph-Ag-Ph metal-organic bonds. (Supplementary Fig. 1h).

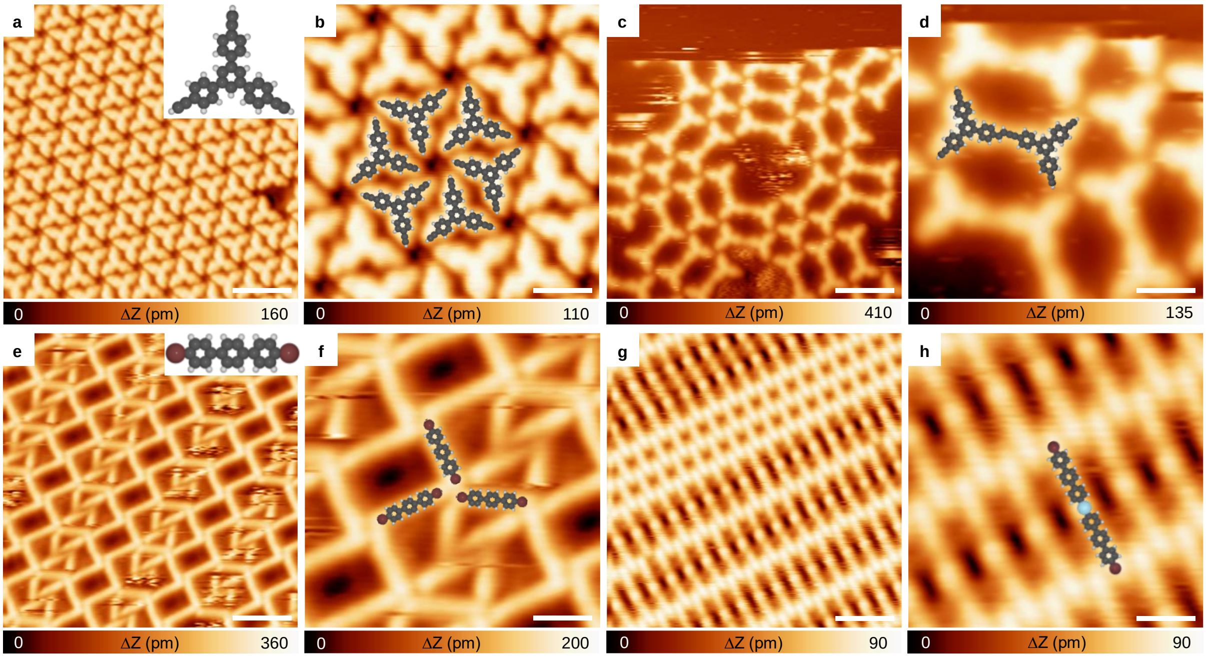

Supplementary Fig. 1 | Reaction behavior of Ext-TEB and DBTP separately on Ag(111). **a**, Self-assembly of Ext-TEB upon deposition on a Ag(111) surface held at RT. The inset gives the structure model of Ext-TEB. **b**, High-resolution STM image of **a**, superposed with the molecular structural model. **c**, Representative STM topographic image after annealing the sample shown in **a** at 400 K for 10 minutes. **d**, Zoomed STM image of **c**, superposed with the molecular structural model. **e**, STM image after depositing DBTP onto Ag(111) surface held at RT. The inset gives the structure model of DBTP. **f**, High-resolution STM image of **e**, superposed with the molecular structural model. **g**, Representative STM topographic image after annealing the sample shown in **e** at 370 K for 10 minutes. **h**, High resolution STM image of **g**, superposed with the molecular structural model. Tunneling parameters are I_t = 100 pA and V_b = -1 V for all the STM images. Ag, Br, C and H atoms are represented by blue, brown, gray, and white circles, respectively. Scale bar: **a**, **c**, **e**, **g** 3 nm, **b**, **d**, **f**, **h** 1.2 nm.

Suppl. Note 2. Large-scale STM image of the alkynyl-Ag network

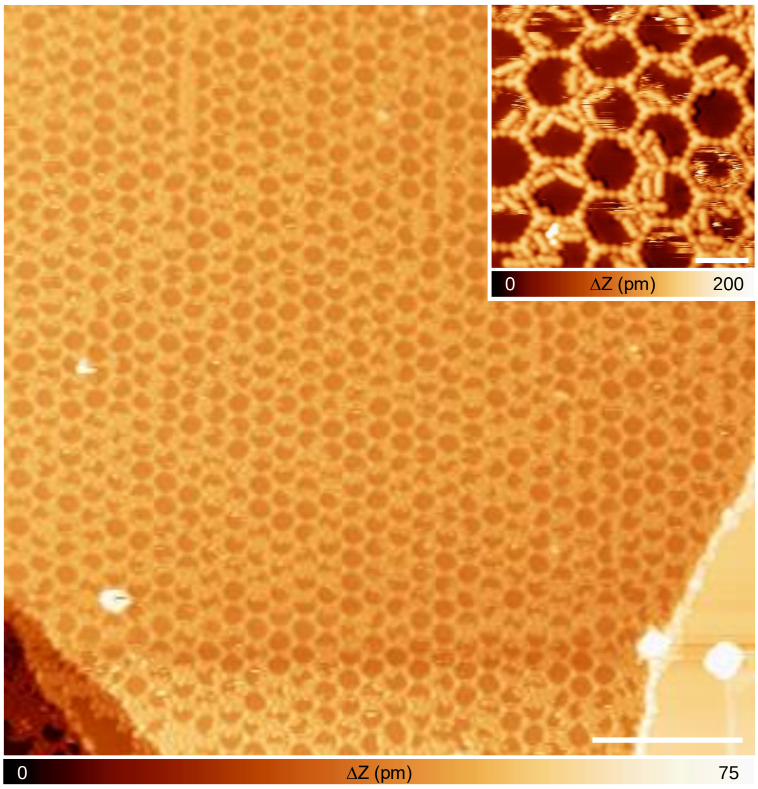

Supplementary Fig. 2 | Formation of alkynyl-Ag network after co-depositing Ext-TEB and DBTP on Ag(111). Large-scale STM image of the alkynyl-Ag network. Insert: zoomed STM image of the alkynyl-Ag network. Tunneling parameters are I_t = 100 pA and V_b = -1 V. Scale bar: 20 nm for the large-scale STM image, 3 nm for the inset.

Suppl. Note 3. Distance measurements for DBTP molecule and its debrominated products

According to previous reports, Br adatoms interact with benzenes via the C-H···Br interaction,[7,8] and Ag adatoms interact with aryl bromides through the Ph-Br···Ag interaction.[9] In both situations, the distance between the adatom and the interacting phenyl ring is much longer than that between Br atom and the adjacent phenyl within a pristine DBTP monomer. It is therefore possible to determine precisely, whether Br atoms are detached by means of distance measurements.

As shown in Supplementary Fig. 3b-g, two distances are measured. The protrusion closer to phenyl groups (0.42 ± 0.1 nm) can be ascribed to Br atoms covalently bond to phenyls. Protrusion further away from phenyl groups (0.59 ± 0.2 nm) can be assigned to Ag adatoms or detached Br adatoms. Consequently, the monomers shown in Supplementary Fig. 3b-d can be attributed to the pristine monomer, monomer with one Br atom detached, and monomer with Br atoms at both sides detached, respectively.

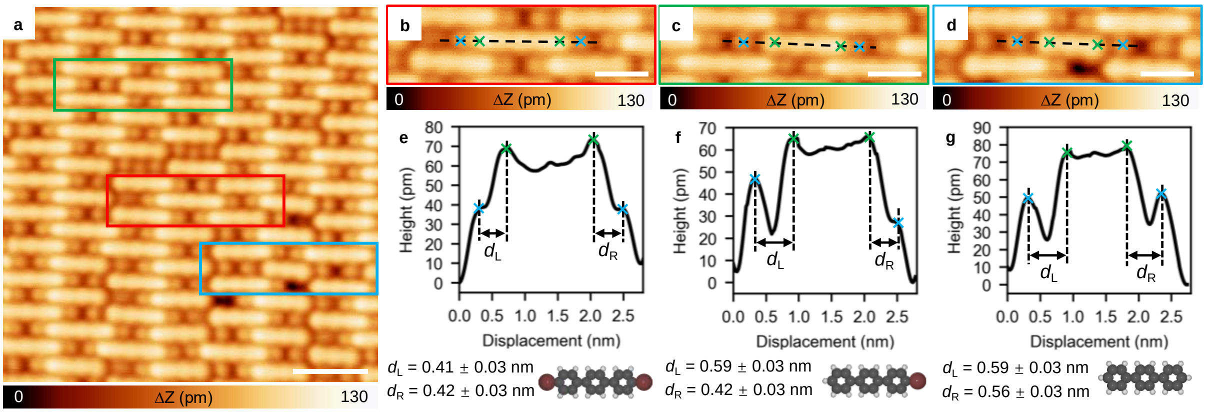

Supplementary Fig. 3 | Distance measurements. a, Self-assembly of DBTP after annealing the sample at 340 K. **b-d**, High resolution STM images of three representative reaction products of DBTP. **e-g**, The corresponding line profiles, measured distances, and structural models. Tunneling parameters are I_t = 100 pA and V_b = -1 V for all the STM images. Br, C, and H atoms are represented by brown, gray, and white circles, respectively. Scale bar: **a** 2 nm, **b**-**c** 0.95 nm.

Suppl. Note 4. Annealing the sample (Ext-TEB + DBTP)/Ag(111) to 370 K

Supplementary Fig. 4a shows a representative highly resolved STM topographic image after annealing the sample shown in Fig. 2 to 370 K. All the DBTP precursors have Br atoms detached. More importantly, all the debrominated phenyl radicals of DBTP are passivated by hydrogen atoms with 100% yield. The corresponding height profile is shown in Supplementary Fig. 4c. The distance between extremal phenylene units is measured to be 0.85 ± 0.10 nm, which is comparable to the length of a monomer, as depicted in Fig. 2k.

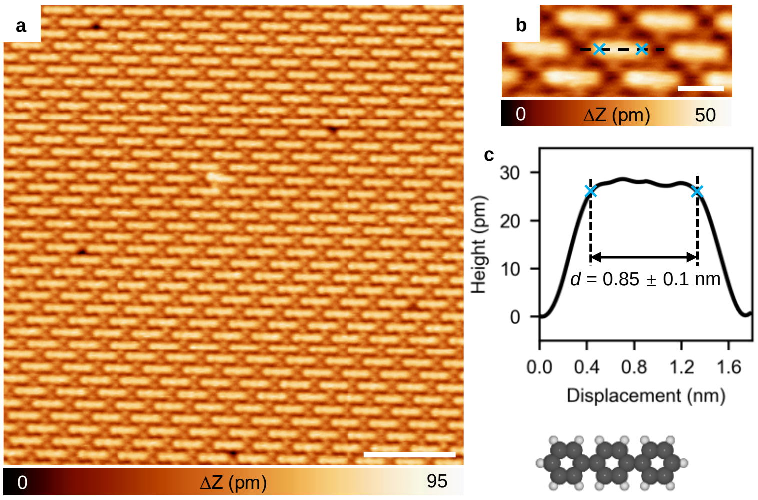

Supplementary Fig. 4 | Passivated DBTP after annealing. a, STM image after annealing the DBTP + Ext-TEB/Ag(111) sample at 370 K. **b**, Zoomed STM image of **a**. **c**, The corresponding line profile and structural model. Tunneling parameters are I_t = 100 pA and V_b = -1 V. C and H atoms are represented by gray and white circles, respectively. Scale bar: **a**, 4 nm, **b**, 1nm.

Suppl. Note 5. Control experiments to confirm H-passivated DBTP

The hydrogen passivation is the key point to support the inter-molecular radical transfer mechanism. We therefore confirmed the hydrogen passivation by complementary control experiments:

1. We further annealed the phase shown in Supplementary Fig. 4 at 420 K (Supplementary Fig. 5) and observe that the self-assembly islands stayed unaffected (annealing at 370 K: a = 1.72 ± 0.03 nm, b = 1.01 ± 0.02 nm, θ = 150 ± 1°; annealing at 420 K: a = 1.69 ± 0.03 nm, b = 1.01 ± 0.02 nm, θ = 150 ± 1°). If the monomers were not passivated, Ullmann-type reactions would have taken place at the elevated temperature.

2. Previous reports demonstrated that the introduction of phenol derivatives can effectively transform the metal-organic oligomers to hydrogen-passivated monomers through the breakage of metal-organic bonds[10]. In this control experiments, we choose 1,3,5-tris(4-hydroxyphenyl)benzene (THPB) as the phenol derivative. Supplementary Fig. 6a shows that DBTP molecules form one-dimensional organometallic chains via Ph-Ag-Ph bonds on Ag(111) after annealing at 400 K. By subsequently depositing THPB molecules on the surface held at 400 K, organometallic wires gradually decompose, resulting in the formation of close-packed assembly islands, as shown in Supplementary Fig. 6b. According to the reference[10], monomers in this assembly island are H-passivated DBTP (the reaction path is schematically illustrated in Supplementary Fig. 6c). The self-assemble structure (a = 1.73 ± 0.03 nm, b = 1.01 ± 0.02 nm, θ = 150 ± 1°) shown in Supplementary Fig. 6b is same to that shown in the Supplementary Fig. 4 and 5.

We therefore believe it is safe to conclude that monomers shown in Supplementary Fig. 4a are passivated (by hydrogen atoms) DBTP monomers.

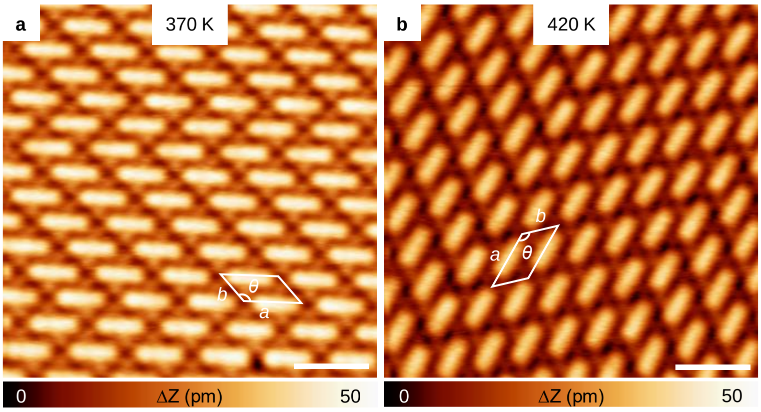

Supplementary Fig. 5 | Thermal annealing for the sample (Ext-TEB + DBTP)/Ag(111). a and **b** Representative STM images after annealing the (Ext-TEB + DBTP)/Ag(111) sample at 370 K and 420 K, respectively. Unit cells are superposed. Tunneling parameters are I_t = 100 pA and V_b = -1 V for all the STM images. Scale bar: **a**, **b** 2 nm.

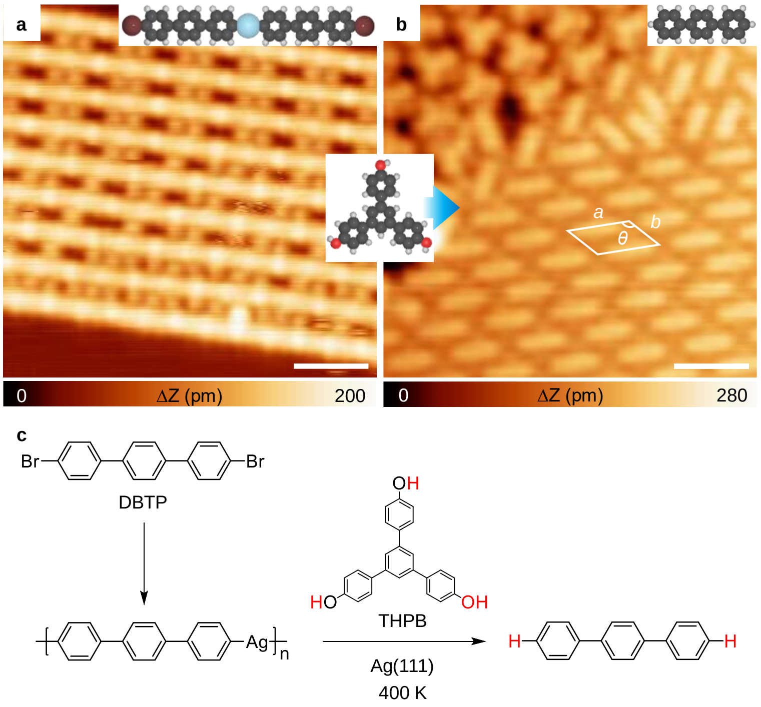

Supplementary Fig. 6 | Structural evolution of DBTP with introducing THPB molecule. a, STM topographic image after depositing DBTP on Ag(111) held at 400 K. **b**, STM topographic image after depositing THPB on the surface shown in **a** held at 400 K. **c**, Scheme of the reaction pathway. Tunneling parameters are I_t = 100 pA and V_b = -1 V for all the STM images. Ag, C, O, Br and H atoms are represented by silver, gray, red, brown, and white circles, respectively. Scale bar: **a**, **b** 2 nm.

Suppl. Note 6. Desorption of reaction products after annealing.

As shown in Supplementary Fig. 7, after further annealing the sample shown in Fig. 2 at 450 K, alkynyl-Ag species desorb significantly. Meanwhile, the coverage of DBTP also decreases, and the remaining molecules aggregate into large-scale islands.

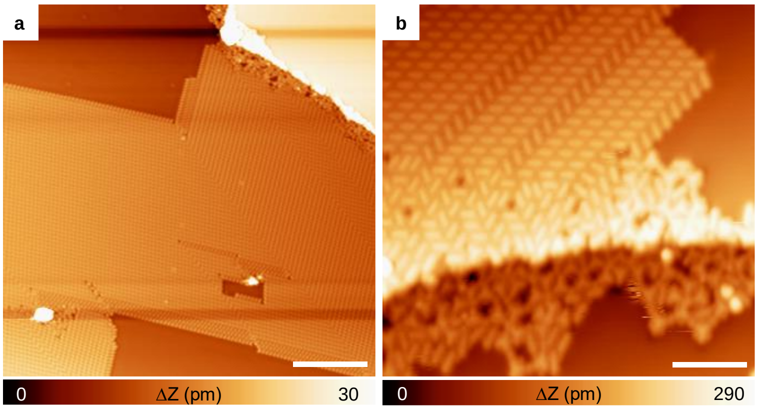

Supplementary Fig. 7 | Desorption of alkynyl-Ag species and H-passivated DBTP molecules after annealing. a Large-scale and **b** zoomed STM images after annealing the DBTP + Ext-TEB/Ag(111) sample at 450 K. Tunneling parameters are I_t = 100 pA and V_b = -1 V for all the STM images. Scale bar: **a**, 20 nm, **b**, 6 nm.

Suppl. Note 7. C 1s TP-XPS mapping

We obtained the C 1s spectra of TP-XPS from 200 to 550 K with a linear heating rate of 0.02 K s^{-1}. In DBTP/Ag(111) and (DBTP + Ext-TEB)/Ag(111) systems (Supplementary Fig. 8a, b), the C 1s core levels exhibit two shoulders with BE at 285 eV and 284.5 eV, respectively. The binding energies (BE) shifting can be ascribed to the work function variation leading by the chemical adsorption of Br adatoms.[9,11-14] On the other hand, in the Ext-TEB/Ag(111) system (Supplementary Fig. 8c), the TP-XPS spectra exhibit a single dominant peak with BE at 285 eV. Furthermore, the intensity of C 1s core levels in the (DBTP + Ext-TEB)/Ag(111) sample decreases dramatically at temperatures that is higher than 450 K, which differs from that shown in Supplementary Fig. 8a, c.[15] This phenomenon agrees well with our STM observations that significant desorption is observed for both the alkynyl-Ag species and passivated phenylenes at elevated temperature. In contrast, Ullmann reactions and coupling reaction of alkynes take place in DBTP/Ag(111) and Ext-TEB/Ag(111) systems, respectively. The formed products hardly desorb from the surface.

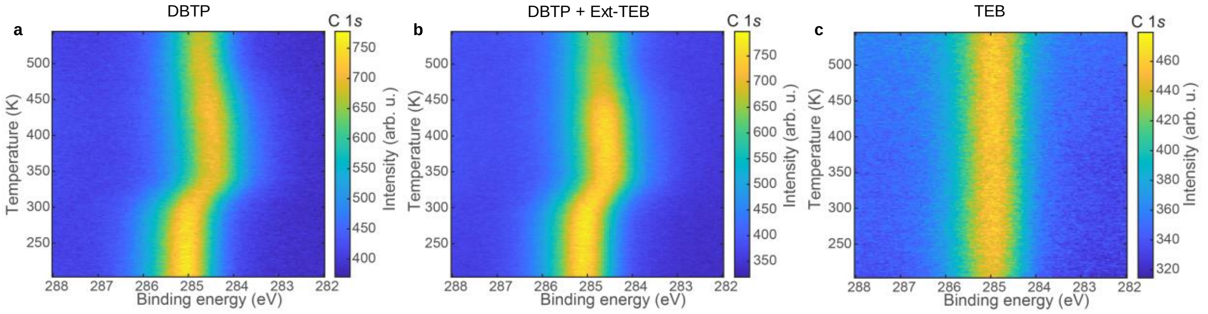

Supplementary Fig. 8 | C 1s TP-XPS data. a-c, C 1s spectra of TP-XPS from 200 to 550 K with a linear heating rate of 0.02 K s^{-1} for DBTP/Ag(111), (DBTP + Ext-TEB)/Ag(111) and Ext-TEB/Ag(111) systems, respectively. Source data are provided as a Source Data file.

Suppl. Note 8. The reaction behavior of Ext-TEB with Br adatoms

We initially investigate the influence of Br adatoms on the C-H activation of Ext-TEB. The Br adatoms is introduced by annealing the DBTP/Ag(111) sample at 500 K.[16] All the debrominated DBTP molecules connect with each other via the Ph-Ph bonds. Subsequently, Ext-TEB molecules are deposited onto the Ag(111) surface (Supplementary Fig. 9a). Zoomed STM images (Supplementary Fig. 9b, c) reveal that Br adatoms (highlighted by brown dots) decorated within the self-assembly islands. We did not observe the alkynyl-Ag-alkynyl linkages after annealing the sample at 400 K, as shown in Supplementary Fig. 9d, e. Instead, enyne products are formed, which is same to that without the presence of aryl halide precursors.[1-4]

DFT calculations were performed to evaluate the C-H activation of Ext-TEB with the presence of Br adatoms. As shown in Supplementary Fig. 9f, g the presence of Br adatom does not facilitate the dehydrogenation of terminal alkyne, resulting in a high energy barrier of 2.13 eV.

The high reaction temperature, the formation of enyne products and the calculated high reaction barrier suggest that the possibility of employing Br adatom as the active center can be ruled out.

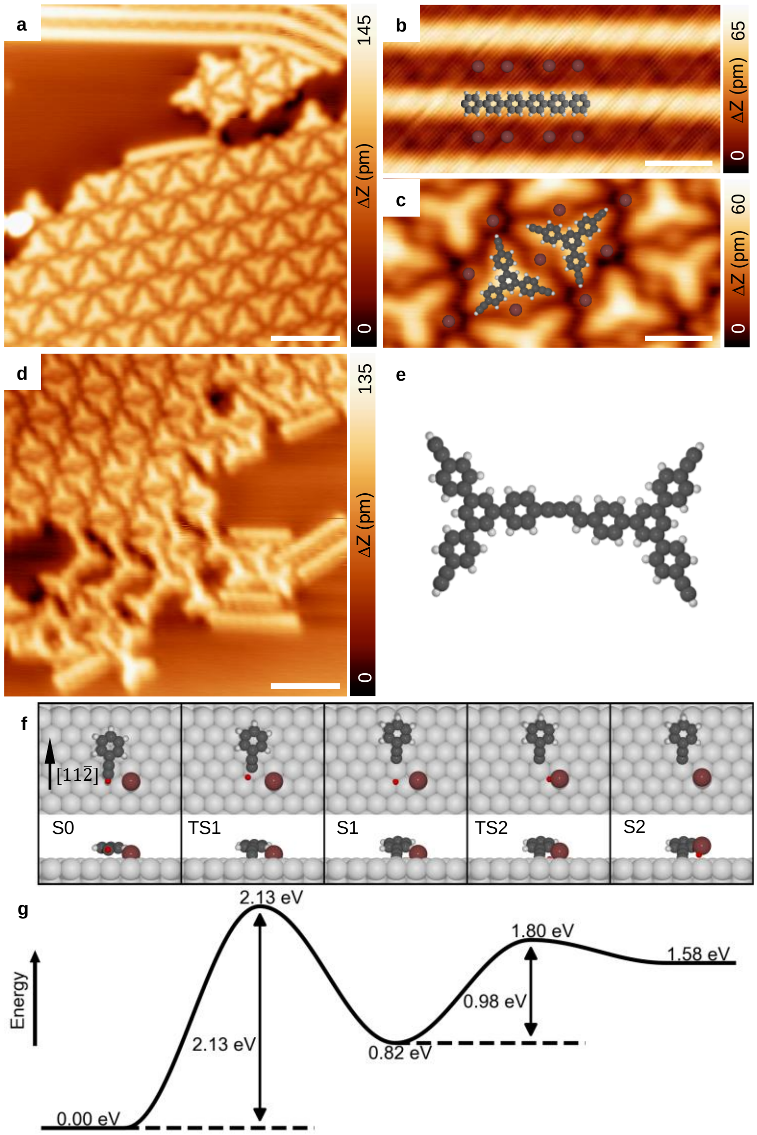

Supplementary Fig. 9 | The reaction behavior of Ext-TEB with Br adatoms. a, self-assembly of Ext-TEB with Br adatom on Ag(111). **b** and **c**, Zoomed-in STM images of **a**. The Br adatoms are highlighted by brown dots. **d**, Formation of enyne products after annealing the sample to 400 K. Corresponding structural model is shown in **e**. **f**, Reaction pathways for the dehydrogenation of phenylacetylene with Br adatom on Ag(111). **g**, Corresponding energy profile is shown in the lower panel. Tunneling parameters are I_t = 100 pA and V_b = -1 V for all the STM images. Ag, Br, C and H atoms are represented by silver, brown, gray, and white circles, respectively. The dissociated H atom in **f** is highlighted by red. Scale bar: **a**, **d** 3 nm, **b**, **c** 1 nm. Source data are provided as a Source Data file.

Suppl. Note 9. The reaction behavior of Ext-TEB with Ag hybrid species

To examine the influence of Ag hybrid species on the dehydrogenation of Ext-TEB, we intentionally prepare Ph-Ag-Ph hybrid species by annealing the DBTP/Ag(111) surface at 400 K.[16] Subsequently, Ext-TEB precursor molecules are deposited onto the surface, as depicted in Supplementary Fig. 10a. High-resolution STM images (Supplementary Fig. 10b, c) confirm the presence of Ag hybrid species. With the participation of Ag hybrid species, the dehydrogenation of terminal alkyne occurs after annealing the Ext-TEB sample at 380 K, which is 40 K higher than that in the bi-component system (Fig. 2). As shown in Supplementary Fig. 10d and e, the dehydrogenated Ext-TEB molecules connect with each other or with DBTP monomer by alkynyl-Ag-alkynyl and alkynyl-Ag-phenyl species.

DFT calculations were carried out to investigate the dehydrogenation of phenylacetylene assisted by Ag hybrid species (Supplementary Fig. 10f, g). Ag hybrid assisted dehydrogenation of phenylacetylene has an energy barrier of 1.20 eV, which is 0.42 eV higher than that with assistance of surface-stabilized phenyl radical (0.78 eV, as shown in Fig. 3a), agrees well with the experimental observations.

The control experiments and the DFT calculations suggest that Ag hybrid species do reduce the dehydrogenation barrier of alkynes, though they are not the active center for the reaction taken place at 340 K in the bi-component systems shown in Fig. 2.

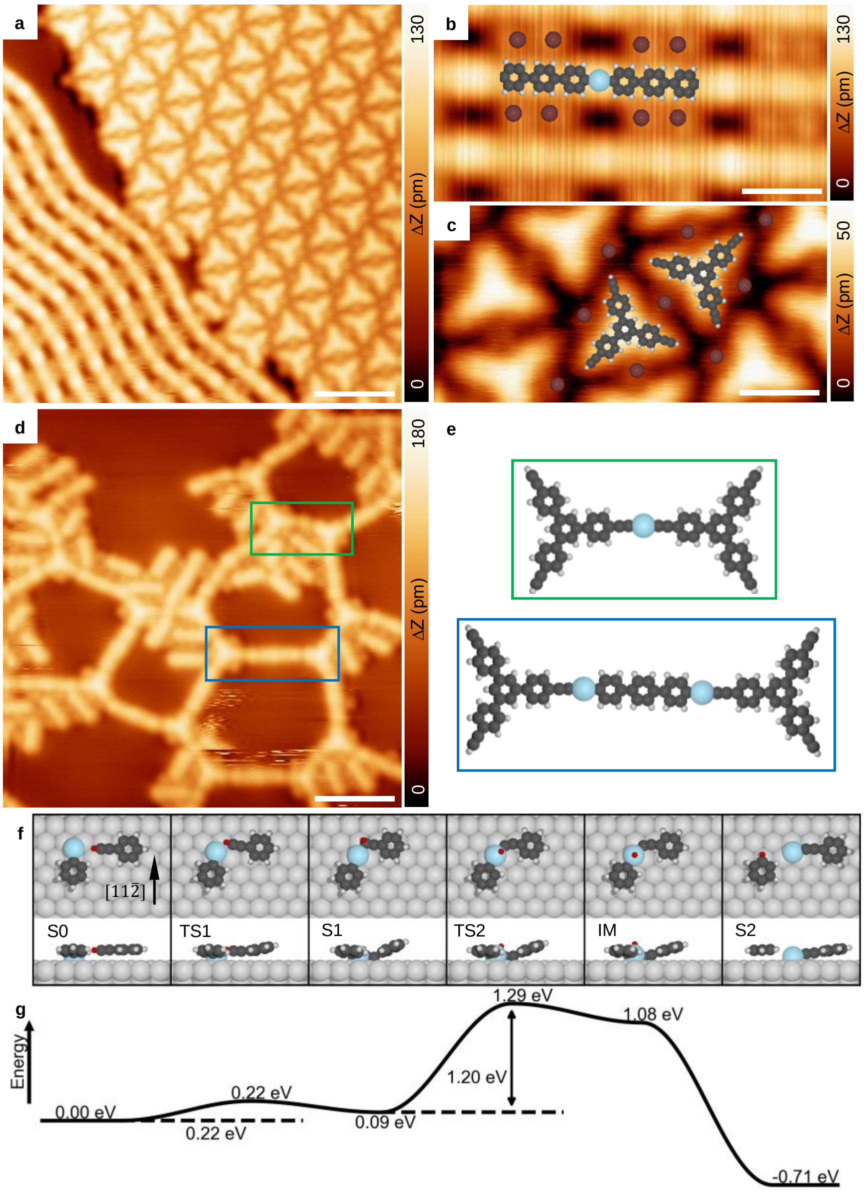

Supplementary Fig. 10 | The reaction behavior of Ext-TEB with Ag hybrid species. a, Large scale STM image after depositing Ext-TEB onto the Ag(111) surface at RT with the presence of Ag hybrid species. **b** and **c**, Zoomed-in STM images of **a**. **d**, The STM image of organometallic products obtained after annealing the sample to 380 K. **e**, Structural models of products marked in panel **d**. **f**, Reaction pathways for the dehydrogenation of phenylacetylene with Ag hybrid species on Ag(111). **g**, Corresponding energy profile is shown in the lower panel. Tunneling parameters are I_t = 100 pA and V_b = -1 V for all the STM images. Ag, hybrid Ag, C and H atoms are represented by silver, blue, gray, and white circles, respectively. The dissociated H atom is highlighted by red. Scale bar: **a**, **d** 3 nm, **b**, **c** 1 nm. Source data are provided as a Source Data file.

Suppl. Note 10. The diffusion of Ext-TEB and DBTP on Ag(111)

The diffusion abilities of precursors are important, as it determines whether the initial states of the DFT calculation shown in Fig. 3 can be achieved. When the radical transfer reaction occurs (340 K), the precursor molecules indeed diffuse sufficiently on Ag(111). Support for this is provided in the following:

1. As shown in Supplementary Fig. 11a and b, fuzzy patterns are observed after depositing submonolayer Ext-TEB (0.2 ML) and DBTP (0.2 ML) separately on Ag(111). The self-assembled islands are stabilized after the coverage increases to about 1 ML (Supplementary Fig. 1). Moreover, after co-depositing 0.2 ML Ext-TEB and 0.2 ML DBTP molecules on Ag(111), fuzzy patterns are obtained at 77 K, as shown in Supplementary Fig. 11c. Increasing the total coverage close to 1 ML leads to the stabilization of the Ext-TEB and DBTP domains (Supplementary Fig. 11d). Despite of that, one can still observe fuzzy areas between adjacent domains. The fuzzy patterns shown in Supplementary Fig. 11 suggest that at 77 K only weak molecule (Ext-TEB/DBTP)-substrate and molecule (Ext-TEB/DBTP)-molecule (Ext-TEB/DBTP) interactions are exerted.

2. A previous study has demonstrated that depositing aryl bromide precursors on Ag(111) leads to the coexistence of self-assemble islands and fuzzy regions at 97 K on Ag(111).[9] The size of fuzzy regions increases significantly after annealing the sample to 113 K, due to the weak halogen bonding.[5,17] Meanwhile, given the weak C-H···π interactions between terminal alkynes,[18,19] the decomposition of Ext-TEB assembly islands at elevated temperatures is foreseeable. Moreover, phenylacetylene molecules diffuse sufficiently below 120 K on Cu(100).[20,21]

It is therefore safe to conclude that both Ext-TEB and DBTP precursor molecules diffuse sufficiently at the reaction temperature (340 K), such that the initial state shown in Fig. 3 can easily be populated.

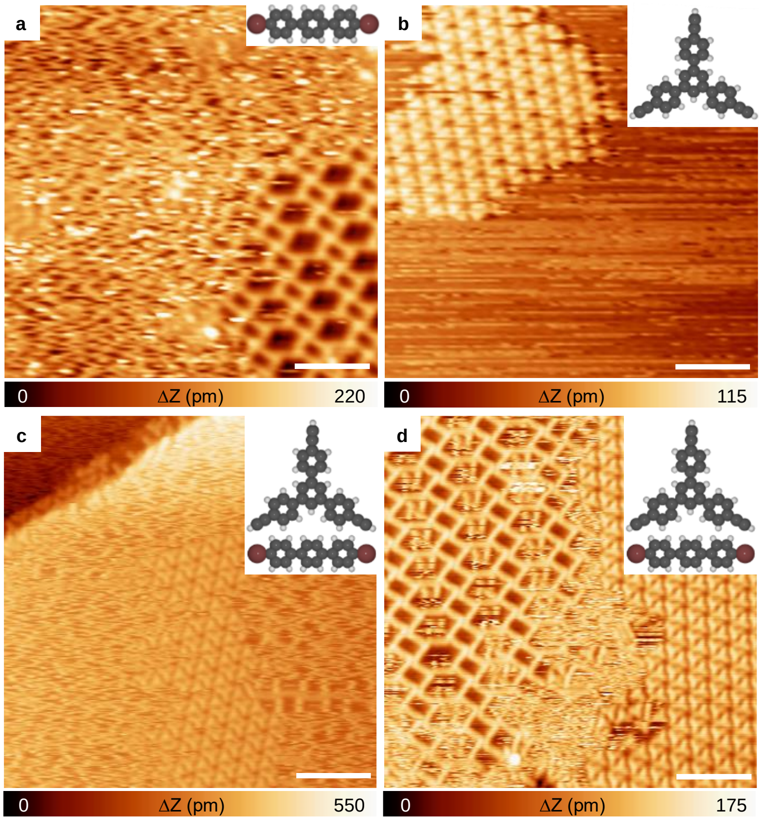

Supplementary Fig. 11 | The sufficient diffusion of Ext-TEB and DBTP molecules on Ag(111). a and **b**, STM topographic images after separately depositing DBTP (0.2 ML) and Ext-TEB (0.2 ML) on Ag(111) held at RT, respectively. **c,** STM topographic images after the co-deposition of 0.2 ML Ext-TEB and 0.2 ML DBTP on Ag(111) held at RT. **d,** STM topographic images after the co-deposition of 0.4 ML Ext-TEB and 0.6 ML DBTP on Ag(111) held at RT. Tunneling parameters are I_t = 100 pA and V_b = -1 V for all the STM images. C, Br and H atoms are represented by gray, brown and white circles, respectively. Scale bar: **a**-**d**, 6 nm.

Suppl. Note 11. Diffusion barriers of halogen atoms on Ag(111)

Supplementary Fig. 12a illustrates the diffusion pathway of the Br atom on Ag(111). The calculated diffusion barrier is 121.26 meV. Similarly, the diffusion barriers for I and Cl atoms on Ag(111) are calculated to be 78.20 meV and 120.68 meV, respectively (Supplementary Fig. 10b).

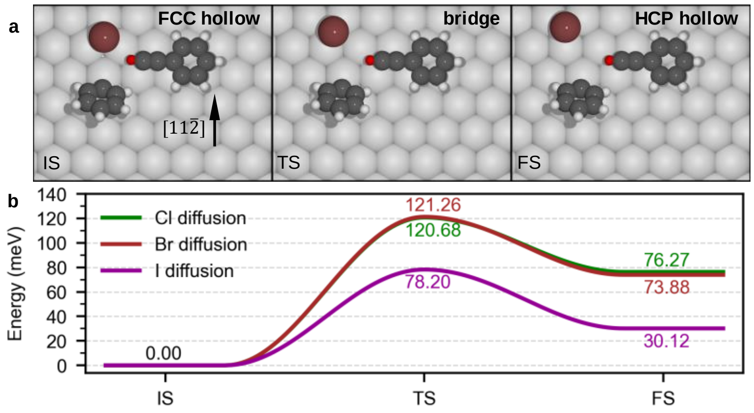

Supplementary Fig. 12 | Diffusion of halogen atoms on Ag(111). a, The calculated diffusion pathway of a Br atom on Ag(111). **b**, The energy profiles for the diffusion of Cl, Br, and I atoms on Ag(111), respectively. Ag, Br, C and H atoms are represented by silver, brown, gray, and white circles, respectively. Source data are provided as a Source Data file.

Suppl. Note 12. The reaction pathway to form the alkynyl-Ag-alkynyl bridging motifs

The formation of alkynyl-Ag-alkynyl motif after the radical transfer process was studied by DFT calculations. As shown in Supplementary Fig. 13a, two isolated alkynyl radicals are incorporated with a silver adatom to form the alkynyl-Ag-alkynyl linkage by overcoming an activation energy of 0.25 eV (Supplementary Fig. 13b). Such energy barrier is much lower than that of debromination of bromobenzene (1.05 eV) and the radical transfer process (0.78 eV). Consequently, the alkynyl-Ag-alkynyl organometallic structure can readily be formed as soon as the radical transfer completes. The low barrier of the formation of alkynyl-Ag-alkynyl motif explains why alkynyl radicals are not observed on Ag(111) experimentally.

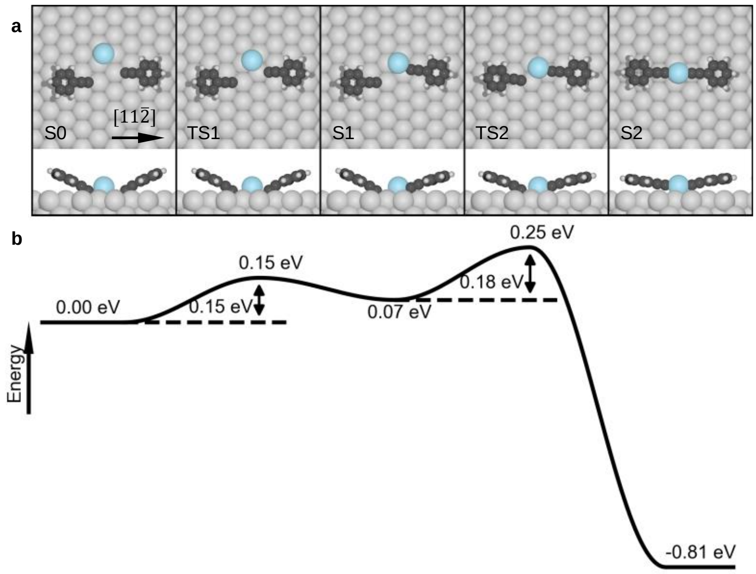

Supplementary Fig. 13 | Formation of alkynyl-Ag-alkynyl bridging motifs on Ag(111). a, The reaction pathways for the formation of alkynyl-Ag-alkynyl linkage on Ag(111). **b**, the corresponding energy profiles. Ag, Ag adatom, C and H atoms are represented by silver, blue, gray, and white circles, respectively. Source data are provided as a Source Data file.

Suppl. Note 13. Reaction behavior after depositing an excess of DBTP molecules in the bi-component system.

Supplementary Fig. 14a gives a representative STM image after co-depositing Ext-TEB and an excess of DBTP molecules on Ag(111) held at RT. The assembled structure of precursor molecules is same as that observed in Fig. 2a. Annealing the sample at 370 K leads to the formation of various oligomers, as shown in Supplementary Fig. 14b and c. All dehydrogenated alkynyl groups interact with phenyl groups via phenyl-Ag-alkynyl bonds due to the excess of DBTP. Meanwhile, phenyl-Ag-phenyl motifs are also observed.

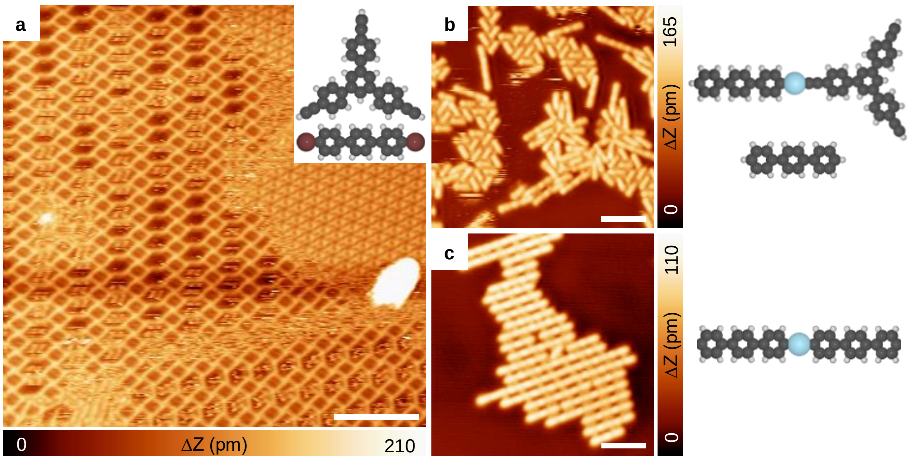

Supplementary Fig. 14 | Reaction behavior with an excess of DBTP. a, Large-scale STM image after co-deposition of Ext-TEB and DBTP on Ag(111) held at RT (DBTP is excess). **b** and **c**, STM topographic images obtained by annealing the sample from RT to 370 K. The corresponding structural models of products are shown in the right panels. Tunneling parameters are I_t = 100 pA and V_b = -1 V for all the STM images. Ag, C and H atoms are represented by blue, gray, and white circles, respectively. Scale bar: **a**, 10 nm, **b**, **c**, 4 nm.

Suppl. Note 14. The reaction behavior of DITP and DCTP on Ag(111)

Deposition of DITP on Ag(111) held at 130 K results in the formation of self-assembled islands (Supplementary Fig. 15a). High-resolution STM image (Supplementary Fig. 15a) suggests the component DITP monomers maintain intact. Deiodination reaction take place by annealing the sample at RT, leading to the formation of one-dimensional organometallic supramolecular structures (Supplementary Fig. 15b; zoomed STM image and the corresponding structural model are provided in the inset).

The situation is quite different for chlorine-substituted precursor molecules (DCTP). Deposition of DCTP on Ag(111) held at RT lead to a well-ordered self-assembled structure, as depicted in Supplementary Fig. 15c. In this phase, each monomer remains intact (the structural model is given in the inset of Supplementary Fig. 15c). DCTP monomers connect to each other via Ph-Ag-Ph bonds after annealing at 450 K (Supplementary Fig. 15d).

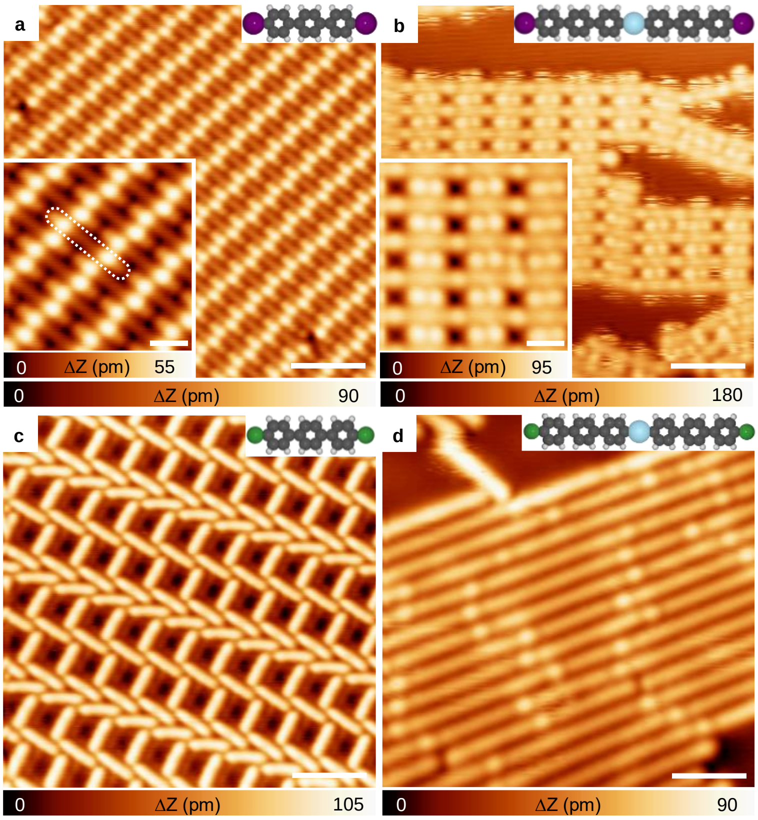

Supplementary Fig. 15 | Reaction behavior of DITP and DCTP on Ag(111). a, STM image after depositing DITP on Ag(111) held at 130 K. The insets give the zoomed-in STM image and the structural model. **b**, STM image acquired by annealing sample shown in **a** at RT. **c**, STM image after deposition of DCTP onto Ag(111) at RT. The insets give the structural model. **d**, Formation of Ph-Ag-Ph bonds after annealing the sample shown in **c** at 450 K. Tunneling parameters are I_t = 100 pA and V_b = -1 V for all the STM images. Ag, C, Cl, H, and I atoms are represented by blue, gray, green, white, and purple circles, respectively. Scale bar: **a**-**d**, 3 nm, inset of **a** and **b**, 1 nm.

Suppl. Note 15. Occurrence of Ullmann reaction for the sample (Ext-TEB + DCTP)/Ag(111)

Supplementary Fig. 16a presents a large scale STM image after annealing the (Ext-TEB + DCTP)/Ag(111) surface at 450 K for 10 minutes. At this stage, the DCTP molecules undergo complete dechlorination reactions, resulting in the formation of organometallic polymers connected by Ph-Ag-Ph bonds, as depicted in Supplementary Fig. 16b.

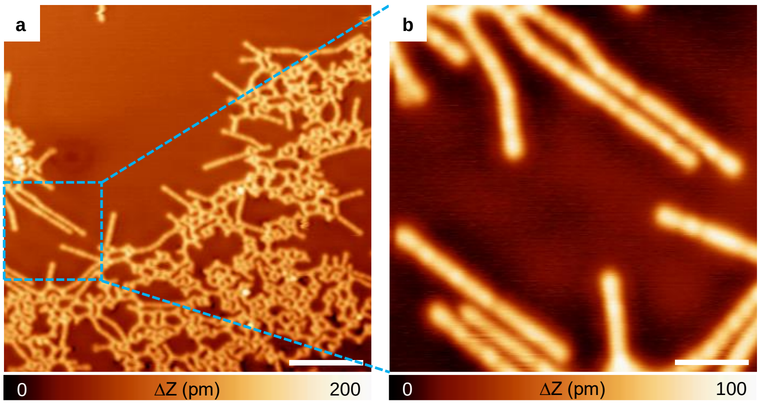

Supplementary Fig. 16 | Ullmann reaction of the (Ext-TEB + DCTP)/Ag(111) sample. a, Large scale STM image after annealing the DCTP + Ext-TEB/Ag(111) surface at 450 K for 10 minutes. **b**, Zoomed-in STM image of the region marked by the dashed square in **a**. Tunneling parameters are I_t = 100 pA and V_b = -1 V for all the STM images. Scale bar: **a**, 10 nm, **b**, 3 nm.

Suppl. Note 16. The reaction pathways for the (phenylacetylene + iodobenzene)/Ag(111) and (phenylacetylene + chlorobenzene)/Ag(111) samples

Supplementary Fig. 17a gives the reaction pathways with iodobenzene and phenylacetylene as the precursor on Ag(111). The corresponding energy profiles are shown in Supplementary Fig. 17b. The energy barrier for the creation of phenyl radical is 0.71 eV, which is slightly lower (0.07 eV) than that for the subsequent radical transfer process.

Supplementary Fig. 17c illustrates the reaction pathways with chlorobenzene and phenylacetylene as the precursor on Ag(111). The corresponding energy profiles are shown in Supplementary Fig. 17d. The calculated activation energy for the dechlorination reaction on Ag(111) is 1.41 eV, which is 0.63 eV higher than that for the subsequent radical transfer process (0.78 eV).

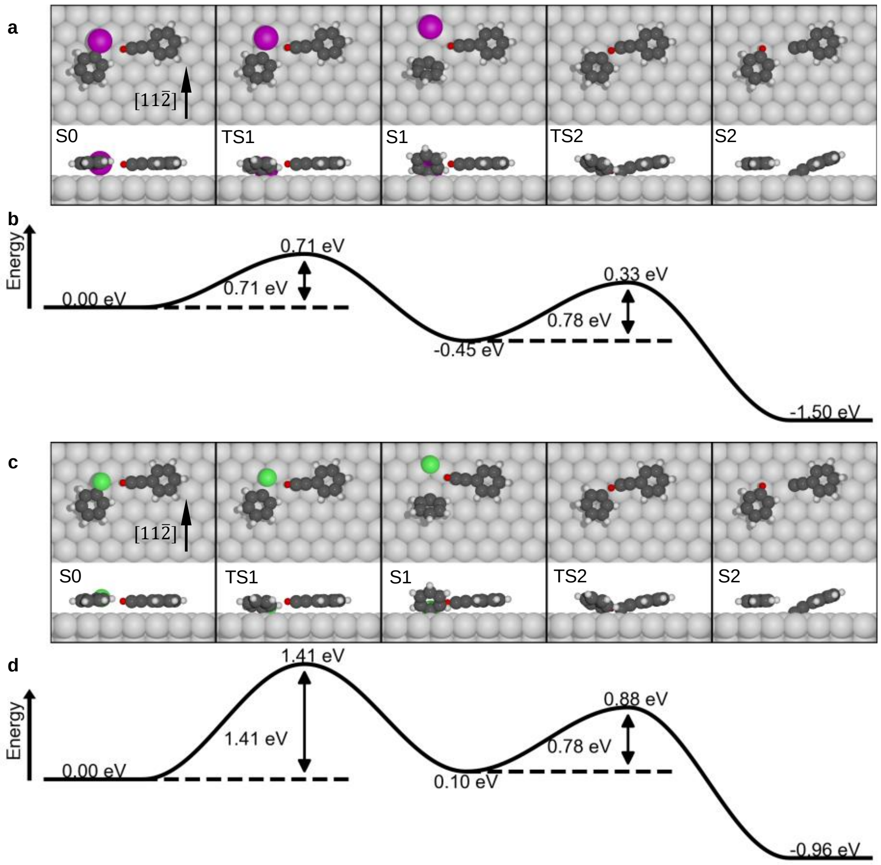

Supplementary Fig. 17 | The reaction pathways for (phenylacetylene + iodobenzene)/Ag(111) and (phenylacetylene + chlorobenzene)/Ag(111) systems. a and **b**, The reaction pathway and the energy profile with iodobenzene and phenylacetylene as the precursor on Ag(111). **c** and **d**, The reaction pathway and the energy profile with chlorobenzene and phenylacetylene as the precursor on Ag(111). Ag, C, H, I and Cl atoms are represented by silver, gray, white, purple and green circles, respectively. The dissociated H atoms are highlighted by red. Source data are provided as a Source Data file.

Suppl. Note 17. TP-XPS mapping of the Br 3d

The Br 3d spectra of TP-XPS are shown in Supplementary Fig. 18.[15] Upon deposition of DBTP onto Ag(111) surface held at 200 K, spin-orbit doublets were observed with Br 3$d_{3/2}$ binding energies (BE) of 70.8 eV on Ag(111) (Supplementary Fig. 18a), indicating the DBTP maintains intact. Starting from 285 K (heating rate is 0.02 K s^{-1}), the Br 3d doublet shifts to lower BE of 67.9 eV (Br 3$d_{3/2}$). This BE shift is attributed to debromination of the precursor. To quantitatively analyze the debromination process, kinetic curves are extracted from the corresponding TP-XPS mapping, as shown in Supplementary Fig. 18b. Similarly, we obtained the kinetic curves of the bi-component system (shown in Supplementary Fig. 18c and d). No distinct difference is observed between their kinetic behavior of debromination reactions. Similar conclusions are drawn by changing the heating rate to 0.01 K s^{-1} and 0.05 K s^{-1}, respectively (Supplementary Fig. 19). This result aligns with the DFT calculations, indicating that the affinity of phenylacetylene to bromobenzene does not lead to reduced debromination barrier (Supplementary Fig. 18e, f).[22]

The TP-XPS measurements and DFT calculations are contradictory to the STM observations that debromination temperature reduces from 370 K in DBTP/Ag(111) to 340 K in (Ext-TEB + DBTP)/Ag(111). Actually, TP-XPS spectra are acquired at elevated temperature during heating, while the STM observations are taken at LN_2 temperature. The debromination of DBTP at elevated temperatures is a dynamic process, even though the phenyl radical may be partially stabilized by Ag(111). Initial debromination of DBTP, therefore, does not entirely transform into the formation of Ph-Ag-Ph bonds after cooling to LN_2 temperature due to the relative low bonding energy of Ph-Ag bonds. On the other hand, in the (Ext-TEB + DBTP)/Ag(111) system, debrominated phenyl radicals are promptly passivated by detached H atoms. Since the newly formed Ph-H bonds are more stable than the Ph-Ag bond, the passivated phenyls maintain during cooling. As a consequence, STM investigations reveal that the hydrogen passivation of DBTP occur at a slightly lower temperature in the bi-component system.

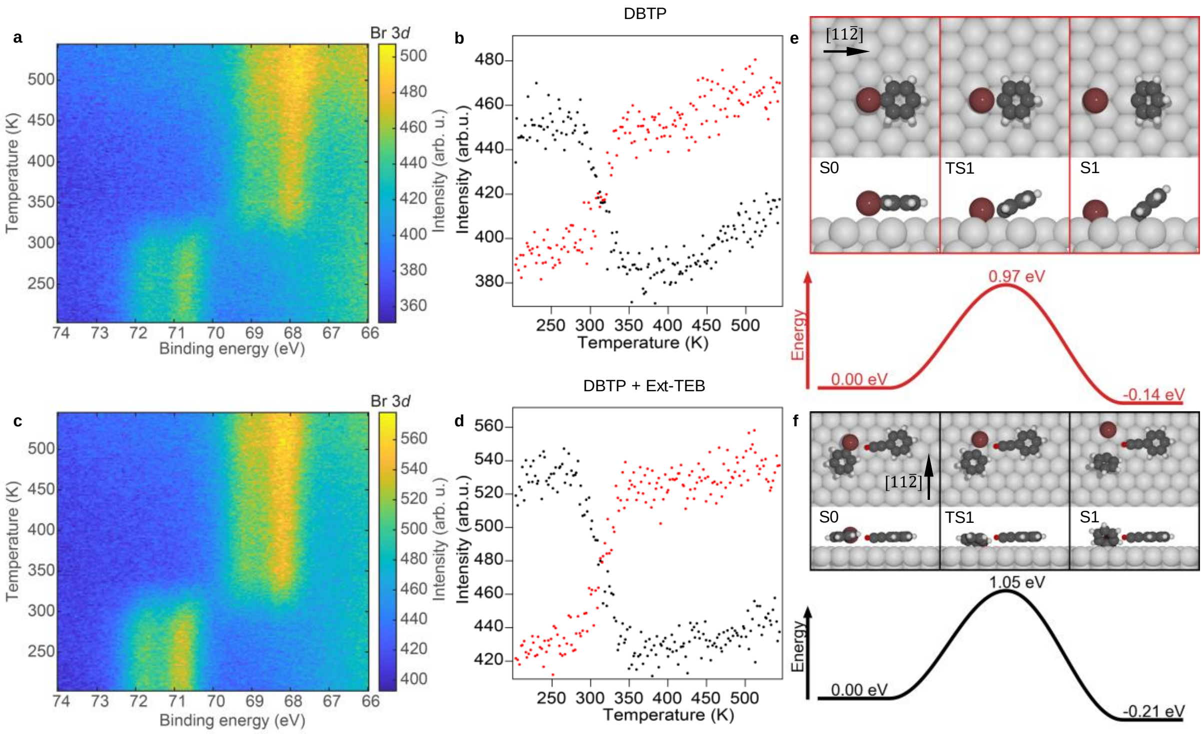

Supplementary Fig. 18 | Br 3d TP-XPS data. a and **c**, Br 3d spectra of TP-XPS acquired from 200 to 550 K with a heating rate of 0.02 K s^{-1}, for DBTP/Ag(111) and (Ext-TEB + DBTP)/Ag(111). **b** and **d**, Corresponding extracted kinetic curves. **e** and **f**, Calculated reaction pathways and the corresponding energy profiles of debromination without and with the affinity of phenylacetylene on Ag(111). Ag, C, H and Br atoms are represented by silver, gray, white and brown circles, respectively. The H dissociated from the phenylacetylene is highlighted by red. Source data are provided as a Source Data file.

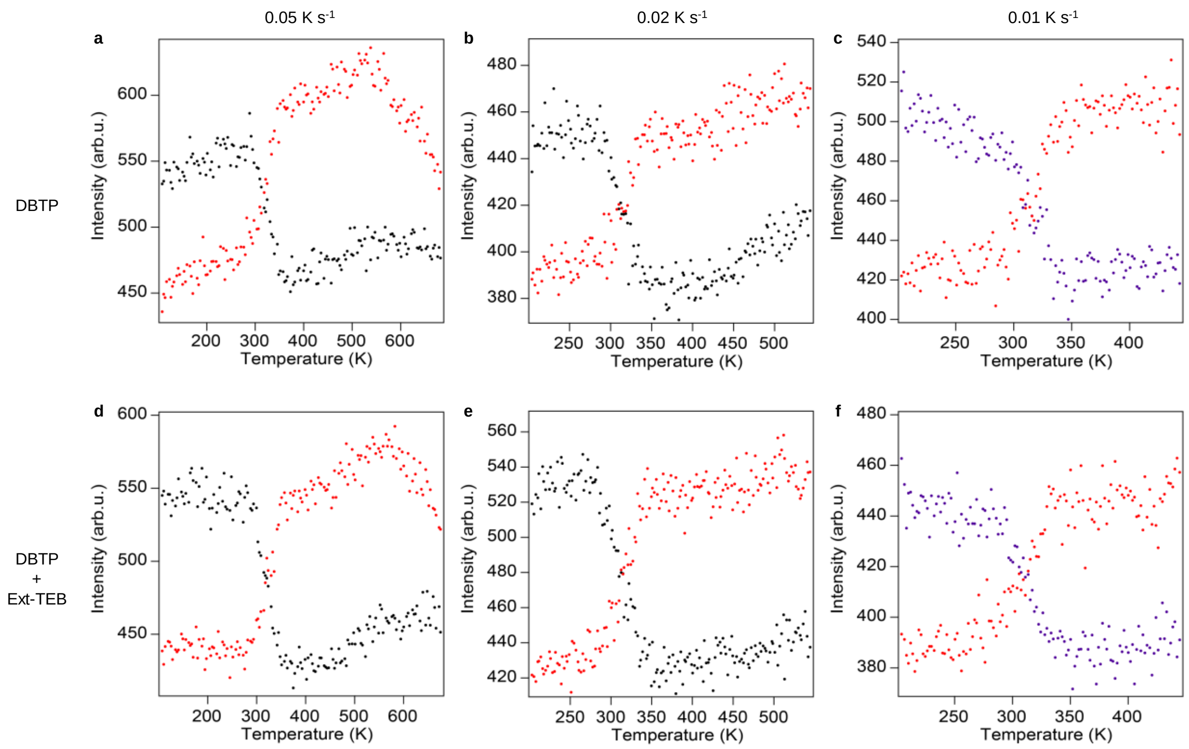

Supplementary Fig. 19 | kinetic behavior of debromination reactions. a-c, Br 3d spectra of TP-XPS with a heating rate of 0.05 K s^{-1}, 0.02 K s^{-1} and 0.01 K s^{-1}, respectively, for the DBTP/Ag(111) systems. **d-f**, Br 3d spectra of TP-XPS with a heating rate of 0.05 K s^{-1}, 0.02 K s^{-1} and 0.01 K s^{-1}, respectively, for the (Ext-TEB + DBTP)/Ag(111) systems. Source data are provided as a Source Data file.

Suppl. Note 18. The universality of the inter-molecular radical transfer reaction

In order to study the generality of the inter-molecular radical transfer mechanism, control experiments are conducted.

Firstly, we choose 4-amino-p-terphenyl (APTP) as precursor molecule (The structural model is shown in the inset of Supplementary Fig. 20a). After depositing APTP on a Cu(111) surface held at RT, fuzzy patterns are observed, indicating the fast movement of adsorbed APTP (Supplementary Fig. 20a). This phenomenon aligns with previous reports that the amino groups remain intact on Cu(111) at RT.[23] Dimer products form after annealing the sample at 370 K for 10 minutes, as seen in Supplementary Fig. 18b, which can be ascribed to the formation of N-Cu-N bonds.[23-25] The structural model is shown in the inset of Supplementary Fig. 20b.

In order to study the influence of aryl bromides on the reaction of amino derivatives, 4'-bromo-1,1'-biphenyl-4-amine (BBPA) is chosen as precursor molecule (The structural model is shown in the inset of Supplementary Fig. 20c). Supplementary Fig. 20c shows a representative STM image after depositing BBPA onto Cu(111) kept at RT. BBPA dimers are observed, indicating the formation of N-Cu-N bonds. Br atoms are observed in-between the dimers, suggesting the detachment of Br atoms from BBPA. Importantly, the Ullmann coupling reactions are prevented, suggesting that the resulting radical sites of BBPA are passivated by hydrogen atoms. The corresponding structural model of the dimer products is shown in the inset of Supplementary Fig. 20d. Both the occurrence of dehydrogenative reaction at RT and the passivation of radical sites suggest the occurrence of the radical transfer reaction.

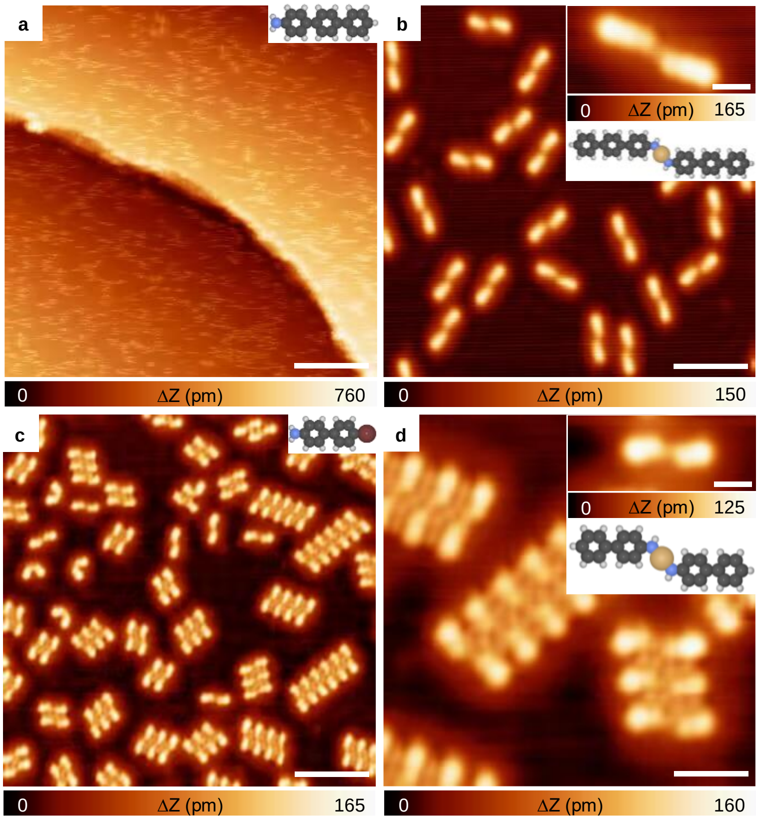

Supplementary Fig. 20 | Reaction behavior of APTP and BBPA on Cu (111). a, STM topographic image after depositing APTP on Cu(111) held at RT. The inset gives the structural model of APTP. **b**, STM topographic image after annealing the surface shown in **a** at 370 K for 10 minutes. Highly resolved STM image and corresponding structural model are shown in the inset. **c**, STM image after depositing BBPA on Cu(111) held at RT. Structural model of BBPA is given in the inset. **d**, High-resolution STM image of **c**. The inset shows the structural model of the dimer product. Tunneling parameters are I_t = 100 pA and V_b = -1 V for all the STM images. Br, C, Cu, N and H atoms are represented by brown, gray, yellow, blue, and white circles, respectively. Scale bar: **a**, 20 nm, **b**, **c**, 6 nm, **d**, 2 nm, inset of **b**, 1nm, inset of **d**, 1 nm.

Suppl. Note 19. The reaction pathway of dehydrogenation reactions of amino derivatives.

Supplementary Fig. 21 presents the calculated reaction pathways of dehydrogenation reactions of phenylamine with and without the assistance of a phenyl radical on Ag(111) and Cu(111), respectively. With the presence of the phenyl radicals, the activation barriers for the detachment of hydrogen atoms are reduced from 1.98 eV to 0.86 eV on Ag(111) and from 1.45 eV to 0.99 eV on Cu(111), respectively.

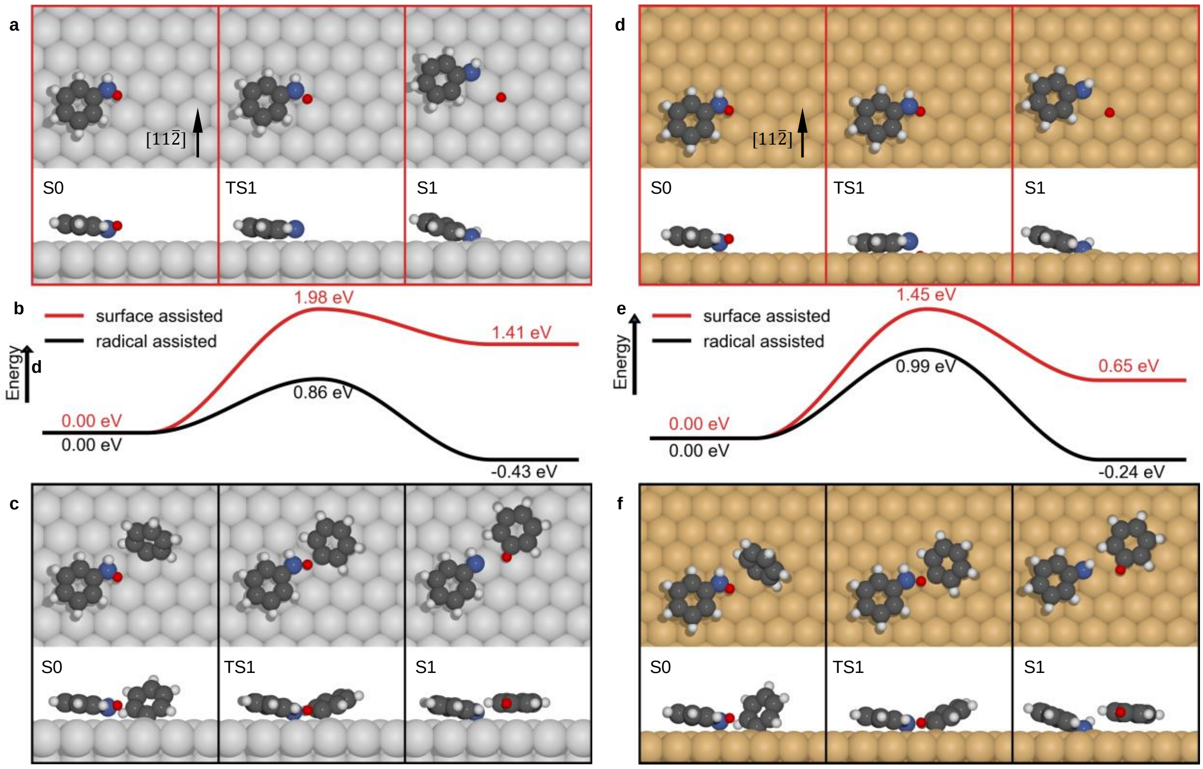

Supplementary Fig. 21 | The reaction pathways of dehydrogenation reactions of phenylamine with and without the assistance of a phenyl radical. a and **c**, Reaction pathways without the presence of phenyl radical and with the presence of phenyl radical on Ag(111). **b**, The corresponding energy profiles. **d** and **f**, Reaction pathways without the presence of phenyl radical and with the presence of phenyl radical on Cu(111). **e**, The corresponding energy profiles. Ag, Cu, C, H, and N atoms are represented by the silver, yellow, gray, white, and blue circles, respectively. H dissociated from the aminobenzene is highlighted by red. Source data are provided as a Source Data file.

Suppl. Discussion 1. Explanations for the high number of Br adatoms in STM images

The observation of the excess of Br adatoms is rather common in Ullmann reactions. However, previous papers did not provide definitive explanations for such phenomenon.[26-28]

In our case, the desorption of molecules in the bi-component system at the reaction temperature is not obvious according to our TP-XPS spectra. Moreover, the adsorption of Br atoms generated in the organic evaporator can be ruled out, as no Br adatoms are detected by solely depositing DBTP on Ag(111) held at RT (see Supplementary Fig. 1e). We therefore believe such phenomenon may arises from one or several of the following reasons: (1) there may exist the self-assembly islands of passivated DBTP without the decoration of Br adatoms, which we did not observe. (2) Plenty of monomers adsorb along the step edges. (3) Small amount of passivated bromobenzene molecules desorb from surface, and the desorption amount is lower than the detection limit of TP-XPS.

Supplementary references

1. Lawrence, J. *et al.* Reassessing alkyne coupling reactions while studying the electronic properties of diverse pyrene linkages at surfaces. *ACS Nano* **15**, 4937-4946 (2021).
2. Zhang, C. *et al.* Chemical identification and bond control of π-skeletons in a coupling reaction. *J. Am. Chem. Soc.* **143**, 9461-9467 (2021).
3. Riss, A. *et al.* Imaging single-molecule reaction intermediates stabilized by surface dissipation and entropy. *Nat. Chem.* **8**, 678-683 (2016).
4. Mohammed, M. S. G. *et al.* Steering alkyne homocoupling with on-surface synthesized metal-organic complexes. *Chem. Commun.* **56**, 8659-8662 (2020).
5. Chung, K.-H. *et al.* Molecular multistate systems formed in two-dimensional porous networks on Ag(111). *J. Phys. Chem. C* **117**, 302-306 (2013).
6. Cirera, B. *et al.* Efficient lanthanide catalyzed debromination and oligomeric length-controlled Ullmann coupling of aryl halides. *J. Phys. Chem. C* **121**, 8033-8041 (2017).
7. Lu, J. *et al.* Construction of two-dimensional chiral networks through atomic bromine on surfaces. *J. Phys. Chem. Lett.* **8**, 326-331 (2017).
8. Lu, J. *et al.* Controllable density of atomic bromine in a two-dimensional hydrogen bond network. *J. Phys. Chem. C* **122**, 25681-25684 (2018).
9. Fan, Q. *et al.* Surface adatom mediated structural transformation in bromoarene monolayers: precursor phases in surface Ullmann reaction. *ACS Nano* **12,** 2267-2274 (2018).
10. Xu, Z., Li, X., Tang, Y. Zhang, H. & Chi, L. Break of metal-organic chains induced by 4,4′ -dihydroxybiphenyl on surfaces. *Chem. J. Chinese Universities* **42**, 1241 (2021).
11. Pham, T. A. *et al.* Comparing Ullmann coupling on noble metal surfaces: on-surface polymerization of 1,3,6,8-tetrabromopyrene on Cu(111) and Au(111). *Chem. Eur. J.* **22,** 5937-5944 (2016).
12. Han, D. *et al.* On-surface synthesis of armchair-edged graphene nanoribbons with zigzag topology. *J. Phys. Chem. C* **124,** 5248-5256 (2020).
13. Zhou, X. *et al.* Steering surface reaction at specific sites with self-assembly strategy. *ACS Nano* **11,** 9397-9404 (2017).
14. Chen, M. *et al.* Combined photoemission and scanning tunneling microscopy study of the surface-assisted Ullmann coupling reaction. *J. Phys. Chem. C* **118,** 6820-6830 (2014).
15. Fritton, M. *et al.* The role of kinetics versus thermodynamics in surface-assisted Ullmann coupling on gold and silver surfaces. *J. Am. Chem. Soc.* **141,** 4824-4832 (2019).
16. Lu, H. *et al.* Dissymmetric on-surface dehalogenation reaction steered by preformed self-assembled structure. *J. Phys. Chem. Lett.* **11,** 1867-1872 (2020).
17. Tschakert, J. *et al.* Surface-controlled reversal of the selectivity of halogen bonds. *Nat. Commun.* **11**, 5630 (2020).

18. Li, Q. *et al.* Supramolecular self-assembly of π-conjugated hydrocarbons via 2D cooperative CH/π interaction. *ACS Nano* **6**, 566-572 (2012).
19. Nishio, M. The CH/ π hydrogen bond in chemistry. Conformation, supramolecules, optical resolution and interactions involving carbohydrates. *Phys. Chem. Chem. Phys.* **13**, 13873-13900 (2011).
20. Li, Q. *et al.* Self-assembly directed one-step synthesis of [4]radialene on Cu(100) surfaces. *Nat. Commun.* **9**, 3113 (2018).
21. Li, Q. *et al.* Self-organized and cu-coordinated surface linear polymerization. *Sci. Rep.* **3**, 2102 (2013).
22. Wang, J. *et al.* Influence of molecular configurations on the desulfonylation reactions on metal surfaces. *J. Am. Chem. Soc.* **144,** 21596-21605 (2022).
23. Li, Q. *et al.* Hierarchical dehydrogenation reactions on a copper surface. *J. Am. Chem. Soc.* **140**, 6076-6082 (2018).
24. Wang, J. *et al.* Constructing and transferring two-dimensional tessellation Kagome lattices via chemical reactions on Cu(111) surface. *J. Phys. Chem. Lett.* **21**, 8151-8156 (2021).
25. Song, L. *et al.* Synthesis of two-dimensional metal-organic frameworks via dehydrogenation reactions on a Cu(111) surface. *J. Phys. Chem. C* **124**, 12390-12396 (2020).
26. Zhang, W. *et al.* Stepwise synthesis of N-Ag-N and C-Ag-C organometallic structures on a Ag(111) surface. *J. Phys. Chem. C* **124**, 16415-16422 (2020).
27. Zhang, X. *et al*. On-surface synthesis of thiophene-containing large-sized organometallic macrocycles on the Ag(111) surface. *J. Phys. Chem. C* **125**, 11454-11461 (2021).
28. Abyazisani, M., MacLeod, J. M. & Lipton-Duffin, J. Cleaning up after the party: removing the byproducts of on-surface Ullmann coupling. *ACS Nano* **13**, 9270-9278 (2019).
